# Supplementary material for: HIVprotI: an integrated web based platform for prediction and design of HIV proteins inhibitors
Source: J Cheminform. 2018 Mar 9;10:12. doi: 10.1186/s13321-018-0266-y (PMC5845081; doi:10.1186/s13321-018-0266-y)
Supplement: Supplementary file 2 — Additional file 2. Source code of HIVProtI web server. [file 13321_2018_266_MOESM2_ESM.zip › HIVprotI_Source-code/hivprotI/weka-3-6-6/doc/weka/attributeSelection/LFSMethods.html]

LFSMethods


---


|  |  |  |
| --- | --- | --- |
| **Overview** \| **Package** \| **Class** \| **Tree** \| **Deprecated** \| **Index** \| **Help** \| **Weka's home** | |  |
| **PREV CLASS**   **NEXT CLASS** | **FRAMES**    **NO FRAMES**     **All Classes** |  |
| SUMMARY: NESTED \| FIELD \| CONSTR \| METHOD | DETAIL: FIELD \| CONSTR \| METHOD |  |


---


## weka.attributeSelection Class LFSMethods

```
java.lang.Object
  weka.attributeSelection.LFSMethods
```

**All Implemented Interfaces:**: RevisionHandler

---

``` public class LFSMethods extends java.lang.Object implements RevisionHandler ```

**Version:**
:   $Revision: 1.3 $

**Author:**
:   Martin Guetlein (martin.guetlein@gmail.com)

---

| **Nested Class Summary** | |
| --- | --- |
| `class` | `LFSMethods.Link2`             Class for a node in a linked list. |
| `class` | `LFSMethods.LinkedList2`             Class for handling a linked list. |


**Constructor Summary**

`LFSMethods()`
  
          empty constructor
methods are not static because of access to inner class Link2 and
LinkedList2


| **Method Summary** | |
| --- | --- |
| `java.util.BitSet` | `floatingForwardSearch(int cacheSize, java.util.BitSet startGroup, int[] ranking, int k, boolean incrementK, int maxStale, Instances data, SubsetEvaluator evaluator, boolean verbose)`             Performs linear floating forward selection ( the stopping criteria cannot be changed to a specific size value ) |
| `java.util.BitSet` | `forwardSearch(int cacheSize, java.util.BitSet startGroup, int[] ranking, int k, boolean incrementK, int maxStale, int forceResultSize, Instances data, SubsetEvaluator evaluator, boolean verbose)`             Performs linear forward selection |
| `java.util.BitSet` | `getBestGroup()` |
| `java.util.BitSet` | `getBestGroupOfSize(int size)` |
| `double` | `getBestMerit()` |
| `int` | `getNumEvalsCached()` |
| `int` | `getNumEvalsTotal()` |
| `java.lang.String` | `getRevision()`             Returns the revision string. |
| `int[]` | `rankAttributes(Instances data, SubsetEvaluator evaluator, boolean verbose)` |

**Methods inherited from class java.lang.Object**

`equals, getClass, hashCode, notify, notifyAll, toString, wait, wait, wait`

**Constructor Detail**

### LFSMethods

```
public LFSMethods()
```

:   empty constructor
    methods are not static because of access to inner class Link2 and
    LinkedList2


**Method Detail**

### getBestGroup

```
public java.util.BitSet getBestGroup()
```

:   **Returns:**: best group found by forwardSearch/floatingForwardSearch

---


### getBestMerit

```
public double getBestMerit()
```

:   **Returns:**: merit of best group found by forwardSearch/floatingForwardSearch

---


### getBestGroupOfSize

```
public java.util.BitSet getBestGroupOfSize(int size)
```

:   **Returns:**: best group of size found by forwardSearch

---


### getNumEvalsCached

```
public int getNumEvalsCached()
```

:   **Returns:**: number of cached / not performed evaluations

---


### getNumEvalsTotal

```
public int getNumEvalsTotal()
```

:   **Returns:**: number totally performed evaluations

---


### rankAttributes

```
public int[] rankAttributes(Instances data,
                            SubsetEvaluator evaluator,
                            boolean verbose)
                     throws java.lang.Exception
```

:   **Returns:**: ranking (integer array) of attributes in data with evaluator (sorting is NOT stable!) **Throws:**: `java.lang.Exception`

---


### forwardSearch

```
public java.util.BitSet forwardSearch(int cacheSize,
                                      java.util.BitSet startGroup,
                                      int[] ranking,
                                      int k,
                                      boolean incrementK,
                                      int maxStale,
                                      int forceResultSize,
                                      Instances data,
                                      SubsetEvaluator evaluator,
                                      boolean verbose)
                               throws java.lang.Exception
```

:   Performs linear forward selection

    :   **Parameters:**: `cacheSize` - chacheSize (times number of instances) to store already evaluated sets: `startGroup` - start group for search (can be null): `ranking` - ranking of attributes (as produced by rankAttributes), no ranking would be [0,1,2,3,4..]: `k` - number of top k attributes that are taken into account: `incrementK` - true -> fixed-set, false -> fixed-width: `maxStale` - number of times the search proceeds even though no improvement was found (1 = hill-climbing): `forceResultSize` - stopping criteria changed from no-improvement (forceResultSize=-1) to subset-size: `data` -: `evaluator` -: `verbose` - **Returns:**: BitSet, that cotains the best-group found **Throws:**: `java.lang.Exception`

---


### floatingForwardSearch

```
public java.util.BitSet floatingForwardSearch(int cacheSize,
                                              java.util.BitSet startGroup,
                                              int[] ranking,
                                              int k,
                                              boolean incrementK,
                                              int maxStale,
                                              Instances data,
                                              SubsetEvaluator evaluator,
                                              boolean verbose)
                                       throws java.lang.Exception
```

:   Performs linear floating forward selection
    ( the stopping criteria cannot be changed to a specific size value )

    :   **Parameters:**: `cacheSize` - chacheSize (times number of instances) to store already evaluated sets: `startGroup` - start group for search (can be null): `ranking` - ranking of attributes (as produced by rankAttributes), no ranking would be [0,1,2,3,4..]: `k` - number of top k attributes that are taken into account: `incrementK` - true -> fixed-set, false -> fixed-width: `maxStale` - number of times the search proceeds even though no improvement was found (1 = hill-climbing): `data` -: `evaluator` -: `verbose` - **Returns:**: BitSet, that cotains the best-group found **Throws:**: `java.lang.Exception`

---


### getRevision

```
public java.lang.String getRevision()
```

:   Returns the revision string.

    :   **Specified by:**: `getRevision` in interface `RevisionHandler`
    :   **Returns:**: the revision


---


|  |  |  |
| --- | --- | --- |
| **Overview** \| **Package** \| **Class** \| **Tree** \| **Deprecated** \| **Index** \| **Help** \| **Weka's home** | |  |
| **PREV CLASS**   **NEXT CLASS** | **FRAMES**    **NO FRAMES**     **All Classes** |  |
| SUMMARY: NESTED \| FIELD \| CONSTR \| METHOD | DETAIL: FIELD \| CONSTR \| METHOD |  |


---
